# Unsupervised phenotypic clustering of cardiac MRI data reveals distinct subgroups associated with outcomes in ischemic cardiomyopathy

**DOI:** 10.1007/s10554-025-03541-4

**Published:** 2025-10-23

**Authors:** Gaetano Nucifora, Daniele Muser, Joshua Bradley, Zoi Tsoumani, Giulia De Angelis, Thomas Caiffa, Matthias Schmitt, Gianfranco Sinagra, Chris Miller

**Affiliations:** 1 Cardiac Imaging Unit, Wythenshawe Hospital, Manchester University NHS Foundation Trust, Trust Southmoor Rd, Manchester, M23 9LT UK; 2 Institute of Cardiovascular Sciences, University of Manchester, Manchester, UK; 3 Cardiac Electrophysiology Unit, Department of Biomedical Sciences, Humanitas University, Milan, 20090 Italy; 4 Cardiac Electrophysiology, Cardiovascular Medicine Division, Hospital of the University of Pennsylvania, Philadelphia, PA USA; 5 Royal Brompton and Harefield Hospitals, Guy’s and St Thomas’ NHS Foundation Trust, London, UK; 6 Cardiothoracic Department, Santa Maria della Misericordia University Hospital, Udine, Italy; 7 Institute for Maternal and Child Health-IRCCS “Burlo Garofolo”, Trieste, Italy; 8 Cardiovascular Department, Azienda Sanitaria Universitaria Integrata, Trieste, Italy

**Keywords:** Cardiac magnetic resonance, Ischemic cardiomyopathy, Prognosis, Unsupervised cluster analysis, Machine learning, Artificial intelligence

## Abstract

Ischemic cardiomyopathy (ICM) shows significant heterogeneity in clinical outcomes, challenging traditional risk stratification methods. Cardiac magnetic resonance (CMR) imaging offers detailed insights into myocardial structure and function, yet integrating this multidimensional data remains complex. Aim of the current study was to assess whether unsupervised machine learning could help identify distinct phenotypic subgroups and enhance prognostic accuracy. This study included 319 clinically stable ICM patients. CMR-derived variables, including left ventricular ejection fraction (LVEF), ventricular volumes, and myocardial scar burden, were analysed using KAMILA clustering algorithm. The optimal number of clusters was determined through silhouette analysis, within-cluster sum of squares, and gap statistics. Principal Component Analysis (PCA) visualized the clustering results, and prognostic value was assessed using Cox regression and Kaplan-Meier survival analysis. SHAP (SHapley Additive exPlanations) values were used to evaluate feature importance. Two distinct phenotypic clusters were identified. Cluster 1 (*n* = 219) demonstrated better cardiac function, with higher LVEF, smaller ventricular volumes, and lower scar burden. Cluster 2 (*n* = 100) indicated advanced disease, with lower LVEF, larger volumes, higher scar burden, and greater midwall fibrosis. PCA confirmed clear separation between clusters, explaining 62.6% of the variance. After a median follow-up of 13 months, the composite endpoint was observed in 37 (12%) patients. Patients in Cluster 2 had a significantly higher risk of experiencing the composite outcome (HR = 3.96, *p* < 0.001). SHAP analysis identified ischaemic scar burden, sphericity index, and midwall fibrosis as key predictors of outcomes. Unsupervised clustering of CMR-derived variables identified distinct ICM phenotypes with important prognostic implications. This method improves risk stratification and could help tailor personalised treatment plans, highlighting the potential of machine learning in understanding ICM heterogeneity.

## Introduction

Despite significant advancements in diagnostic and therapeutic strategies, the clinical course of patients with ischemic cardiomyopathy remains highly variable, with notable differences in outcomes such as heart failure progression, arrhythmic events, and mortality [1–3]. This heterogeneity underscores the limitations of traditional risk stratification methods, which often depend on isolated clinical or imaging parameters and do not capture the complex interplay of factors driving disease progression.

Cardiac magnetic resonance (CMR) imaging has become a fundamental tool in the assessment of ICM, offering unmatched insights into myocardial structure, function, and tissue characterisation [4]. CMR-derived variables, such as left ventricular ejection fraction (LVEF) and myocardial scar burden, provide a wealth of quantitative data that can be utilised to better understand disease stages and predict outcomes [5]. Nevertheless, integrating and interpreting these multidimensional data remain challenging, especially within the context of patient heterogeneity.

Unsupervised machine learning techniques, such as clustering, offer a promising approach to tackling this challenge by identifying distinct phenotypic subgroups [6, 7]. By analysing patterns in CMR-derived variables, unsupervised clustering can uncover subtypes of ICM that may indicate differences in disease stage or prognosis. These data-driven phenotypes can improve prognostic accuracy and potentially guide personalised treatment strategies.

In the present pilot study, we aimed to use unsupervised phenotypic clustering of CMR-derived variables to identify distinct subgroups of ICM patients and assess their association with clinical outcomes.

## Methods

### Materials and methods

#### Study population and follow-up

A total of 319 consecutive, clinically stable patients with ICM who had follow-up in the outpatient cardiology clinics of Wythenshawe Hospital and were enrolled in the UHSM CMR Study were included in this analysis. The UHSM CMR Study (ClinicalTrials.gov NCT02326324) is a comprehensive prospective registry of all patients referred for CMR imaging at our institution, approved by the local ethics committee (Greater Manchester West Research Ethics Committee) and conforming to the Declaration of Helsinki; informed consent from all included patients was obtained.

ICM was defined as LV systolic dysfunction with LV ejection fraction (EF) < 50%, assessed by CMR imaging, in the presence of one of the following criteria: (1) clinical history of myocardial infarction, (2) previous percutaneous coronary intervention or coronary artery bypass grafting, (3) angiographic evidence of coronary artery disease with ≥ 70% stenosis in at least one epicardial vessel or ≥ 50% in the left main coronary artery or proximal left anterior descending artery, (4) evidence of ischemic-type LGE explaining the degree of LV systolic dysfunction [8]. Patients within three months of the last episode of decompensated heart failure, with more than mild valvular disease or previous valvular surgery, with an implanted device at the time of CMR imaging, or with poor CMR image quality hampering the assessment of LV volumes and the interpretation of LGE images, were excluded.

Baseline demographic and clinical variables, along with CMR imaging parameters, were prospectively recorded in the registry. Clinical follow-up was conducted by reviewing electronic patient records to determine occurrences such as hospital admissions due to cardiovascular causes, coronary revascularisations (percutaneous coronary intervention or coronary artery bypass grafting), or the implantation of an implantable cardiac defibrillator (ICD). ICD interrogations were reviewed to evaluate ICD therapies among recipients. Cardiovascular mortality was also assessed and defined as death resulting from acute myocardial infarction, heart failure, or arrhythmic events.

The study outcome consisted of a composite endpoint that included: (1) cardiovascular death; (2) aborted sudden cardiac death, defined as resuscitated cardiac arrest due to ventricular fibrillation or haemodynamically unstable ventricular tachycardia; (3) appropriate ICD therapy for ventricular tachycardia or ventricular fibrillation; (4) occurrence of heart failure hospitalisations; and (5) implantation of a left ventricular assist device or occurrence of a heart transplant.

Only patients with complete data for all variables were included in this study.

#### Cardiac magnetic resonance acquisition protocol

CMR studies were performed on 1.5T or 3 T scanners (Avanto and Skyra, Siemens Medical Solutions, Erlangen, Germany) with a cardiac phased-array receiver surface coil and electrocardiogram gating. Three standard cine long-axis slices and a stack of contiguous 8-mm thick cine short-axis slices from the atrioventricular ring to the apex were acquired using a steady-state free precession pulse sequence. LGE images were acquired using the same slice coverage as long-axis and short-axis cine images 15 min after 0.2 mmol/kg intravenous injection of gadolinium-based contrast agent (gadoterate meglumine, Dotarem^®^, Guerbet, France) using an inversion-recovery gradient-echo pulse sequence, individually adjusting inversion time to optimise nulling of apparently normal myocardium, and a phase-sensitive inversion recovery sequence.

#### Cardiac magnetic resonance data analysis

All CMR studies were analysed offline using dedicated software (Circle CVI-42, Circle Cardiovascular Imaging, Calgary, Canada). LV volumes, function, and LV mass were measured using the standard volumetric technique from the cine short-axis images [9]. Volume and mass measurements were indexed to body surface area. The LV sphericity index was also calculated as the ratio of the LV end-diastolic volume (EDV) to the volume of a sphere with the diameter of the LV end-diastolic long-axis from a 4-chamber cine image [10].

The presence or absence of LGE was qualitatively assessed for each LV myocardial segment using the 17-segment cardiac model by reviewing all short- and long-axis contrast-enhanced images [11]; regions with elevated signal intensity had to be confirmed in two spatial orientations. Patterns of LGE were visually classified as either ischemic-type (sub-endocardial or transmural, when occupying ≥ 75% of LV wall thickness) or mid-wall [12]. The extent of ischemic-type and mid-wall LGE was quantitatively determined using the full-width half-maximum technique, as previously described [13, 14]. The extent of LGE was expressed as a percentage of the LV mass (%LV LGE).

#### Statistical methods

All statistical analyses were conducted using R software version 4.3.3 (R Foundation for Statistical Computing, Vienna, Austria). Cluster analysis was performed to identify distinct patient subgroups. The analysis included both continuous variables (age, LVEF, LV EDVi, LV ESVi, LV Massi, Sphericity Index, and ischemic scar expressed as % of LV mass) and categorical variables (gender and presence of midwall scar). The KAMILA (KAy-means for MIxed LArge data) algorithm was implemented with the ‘kamila’ package, which enables simultaneous handling of continuous and categorical data. This method was specifically selected for its capacity to manage mixed data types while maintaining the data’s native scale, integrating k-means for continuous variables with k-modes for categorical variables. The optimal number of clusters was identified using multiple validation methods: silhouette analysis (cluster package), within-cluster sum of squares, and gap statistics. The silhouette width, ranging from − 1 to 1, was computed to evaluate clustering quality, with higher values indicating more distinctly defined clusters. Principal Component Analysis (PCA) was conducted to visualise the clustering results in a reduced-dimensional space. The first two principal components explained 62.6% of the total variance (PC1: 46.22%, PC2: 16.38%), providing a robust representation of the data structure. The final two-cluster solution was chosen based on the highest silhouette score (0.283), optimal cluster size distribution (n₁ = 219, n₂ = 100), and clinically meaningful separation as visualised in the PCA space.

Statistical analyses were conducted to compare the high-risk and low-risk clusters. Continuous variables were compared using the Wilcoxon rank-sum test because of their non-normal distribution, while categorical variables were analysed with the Chi-squared test. P-values below 0.05 were regarded as statistically significant. A radiant plot (radar chart) was generated using the fmsb package to visualise the multidimensional characteristics of the identified clusters. This approach allows for simultaneous visualisation of multiple variables across clusters while accounting for different variable types and scales.

The prognostic value of the identified clusters was assessed through univariate Cox proportional hazards regression analysis; Kaplan-Meier curves were generated using the ‘survival’ package to visualise survival differences between clusters, and the log-rank test was performed to evaluate statistical significance. The composite outcome was also examined using a competing risks framework, treating non-cardiovascular (non-CV) death as a competing event. Cumulative incidence functions (CIFs) were estimated for the composite outcome across clusters, accounting for the competing risk of non-CV death. The subdistribution hazard was modelled using Fine–Gray competing risks regression with the cmprsk package to estimate subdistribution hazard ratios (SHRs) and their 95% confidence intervals, with the cluster as the primary covariate. Event time was defined as the earliest recorded time to either the composite outcome or non-CV death; participants without either event were censored at the latest follow-up time available. SHAP (SHapley Additive exPlanations) values were computed using the shapviz package to assess feature importance in predicting the composite outcome. The analysis incorporated both the binary composite outcome and time-to-event data. Features were normalised to ensure comparability. An XGBoost model was trained using a binary logistic objective function, with parameters including a learning rate (η = 0.1), a maximum tree depth of 3, and sampling rates of 0.8 for both observations and features. SHAP values were calculated to quantify each feature’s contribution to the predicted risk of the composite outcome. Feature importance was ranked based on mean absolute SHAP values, and visualisation was performed using the ggplot2 package to display both the magnitude and direction of feature effects.

## Results

**Fig. 1:**
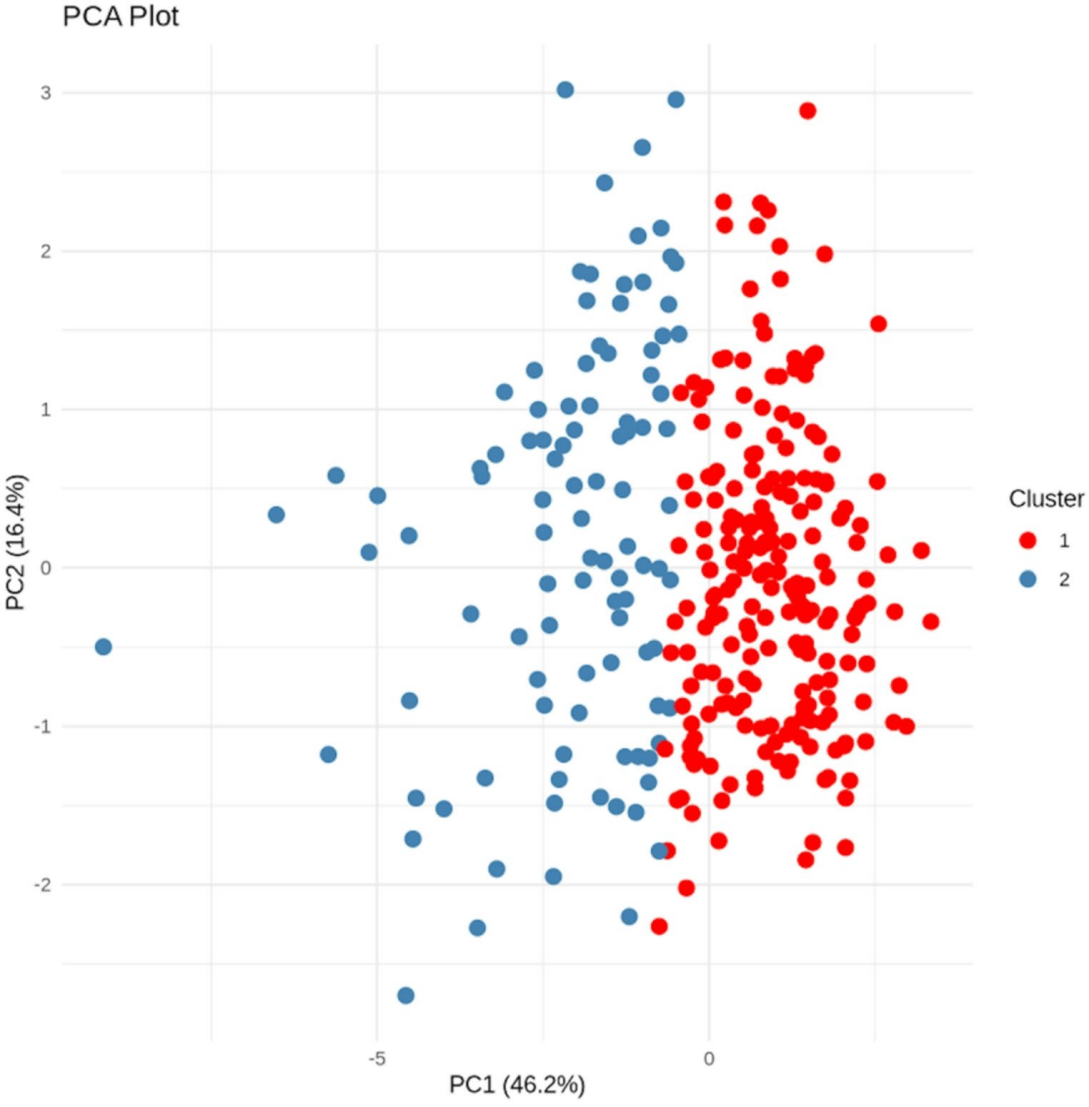
Principal Component Analysis (PCA) Plot of Patient Clusters. This plot shows the results of the unsupervised clustering in two dimensions. Each point represents a patient, coloured according to their assigned cluster (Cluster 1 in red, Cluster 2 in blue). The clear spatial separation between the two coloured groups illustrates the phenotypic differences identified by the clustering algorithm. The first two principal components (PC1 and PC2) together explain 62.6% of the total variance in the dataset

**Fig. 2:**
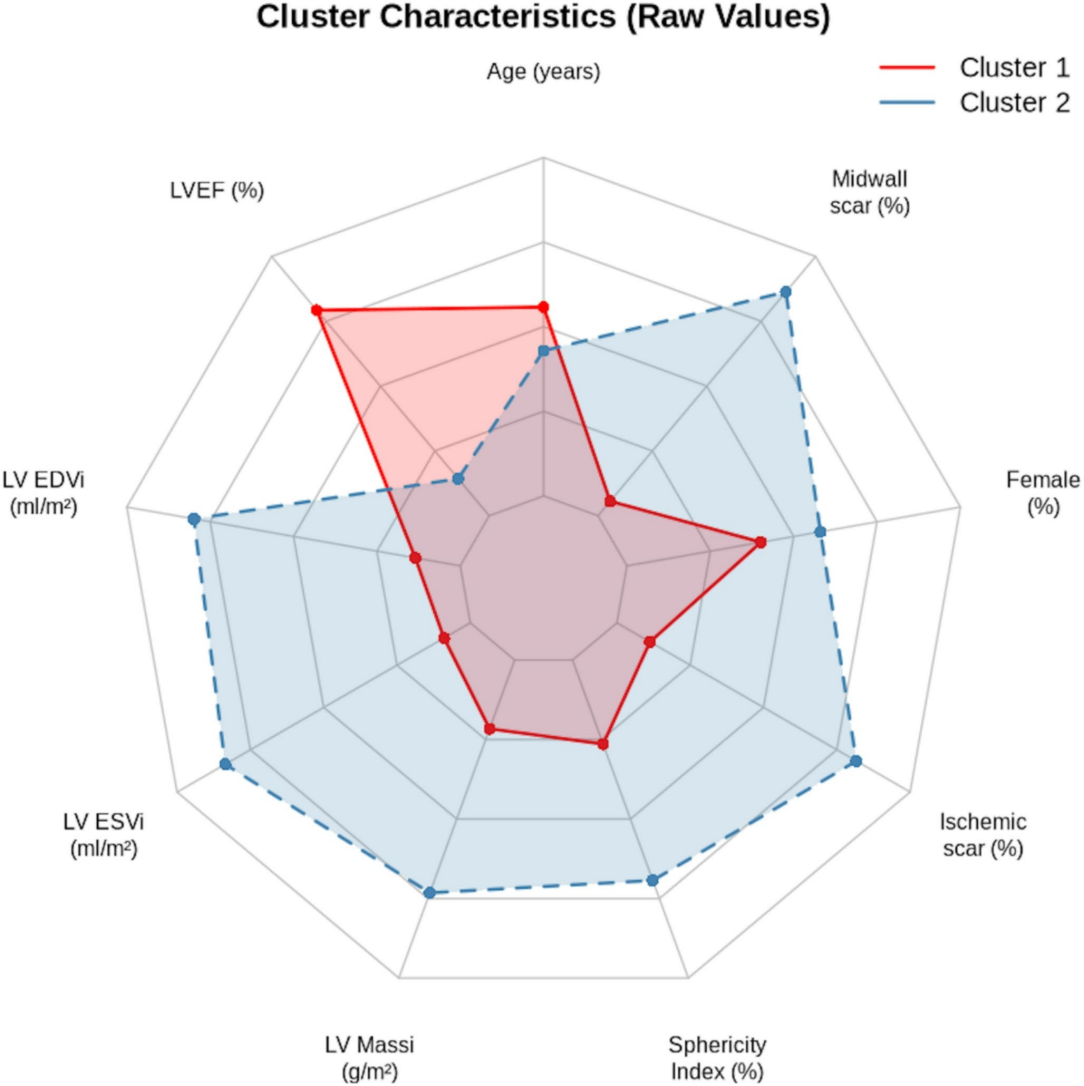
Radar chart of clinical and CMR parameters across identified clusters. This chart provides a comparative overview of the two identified clusters across key variables. Each axis represents a different parameter, which has been normalised to a common scale for comparison. The shape formed by connecting the values for each cluster demonstrates its unique phenotypic signature. Cluster 1 (red) indicates a profile of better-preserved cardiac structure and function, while Cluster 2 (blue) shows a pattern of adverse remodelling and extensive scarring

KAMILA clustering identified two distinct phenotypes among 319 patients with ICM. Cluster 1 (*n* = 219) included patients with better cardiac function, whereas Cluster 2 (*n* = 100) displayed features of more advanced disease. The clusters significantly differed in multiple cardiac CMR parameters (all *p* < 0.001), except for age (*p* = 0.054) and gender distribution (*p* = 0.187) (Table 1). Cluster 1 was characterised by a higher left ventricular ejection fraction (43.0 ± 6.3% vs. 28.0 ± 7.3%), smaller indexed left ventricular end-diastolic volume (96.7 ± 16.3 vs. 139.0 ± 29.0 mL/m²), smaller indexed end-systolic volume (56.2 ± 11.6 vs. 97.1 ± 28.3 mL/m²), and lower indexed LV mass (65.9 ± 11.4 vs. 78.1 ± 19.2 g/m²). Notably, Cluster 2 exhibited a higher prevalence of midwall fibrosis (17.3% vs. 6.8%, *p* = 0.007) and a greater sphericity index (52.0 ± 11.4 vs. 45.6 ± 11.1, *p* < 0.001). Principal component analysis (Fig. 1) revealed a clear separation between the two clusters, with the first two principal components accounting for 62.6% of the overall variance (PC1: 46.2%, PC2: 16.4%). This substantial proportion of explained variance supports the robustness of the identified phenotypes. The radar chart analysis (Fig. 2) underscored the multidimensional nature of the differences between clusters.

**Table 1:** Differences in age, gender and CMR characteristics between the two clusters

Variable	Cluster 1 (*n* = 219)	Cluster 2 (*n* = 100)	*P* value
Age	67.0 ± 11.2	63.5 ± 11.3	0.054
Male gender	186 (85%)	91 (91%)	0.187
LVEF (%)	43.0 ± 6.3	28.0 ± 7.3	< 0.001
LV EDVi (mL/m^2^)	96.7 ± 16.3	139.0 ± 29.0	< 0.001
LV ESVi (mL/m^2^)	56.2 ± 11.6	97.1 ± 28.3	< 0.001
LV Massi (g/m^2^)	65.9 ± 11.4	78.1 ± 19.2	< 0.001
Sphericity Index	45.6 ± 11.1	52.0 ± 11.4	< 0.001
Ischemic scar (% of LV)	12.9 ± 7.6	24.2 ± 9.5	< 0.001
Midwall scar	15 (7%)	17 (17%)	0.007

After a median follow-up of 13 (6–28) months, 84 patients underwent coronary revascularisation procedures (45 patients had a percutaneous coronary intervention, and 39 patients had coronary artery bypass grafting), and 59 (19%) patients underwent ICD implantation. The composite endpoint was recorded in 37 (12%) patients; when analysing the components of the composite outcome separately, cardiovascular death was recorded in 12 (4%) patients, aborted SCD in 3 (1%), appropriate ICD interventions in 7 (2%), heart failure hospitalisations in 19 (6%), LVAD implantation in 3 (1%), and heart transplantation in 2 (1%).

**Fig. 3:**
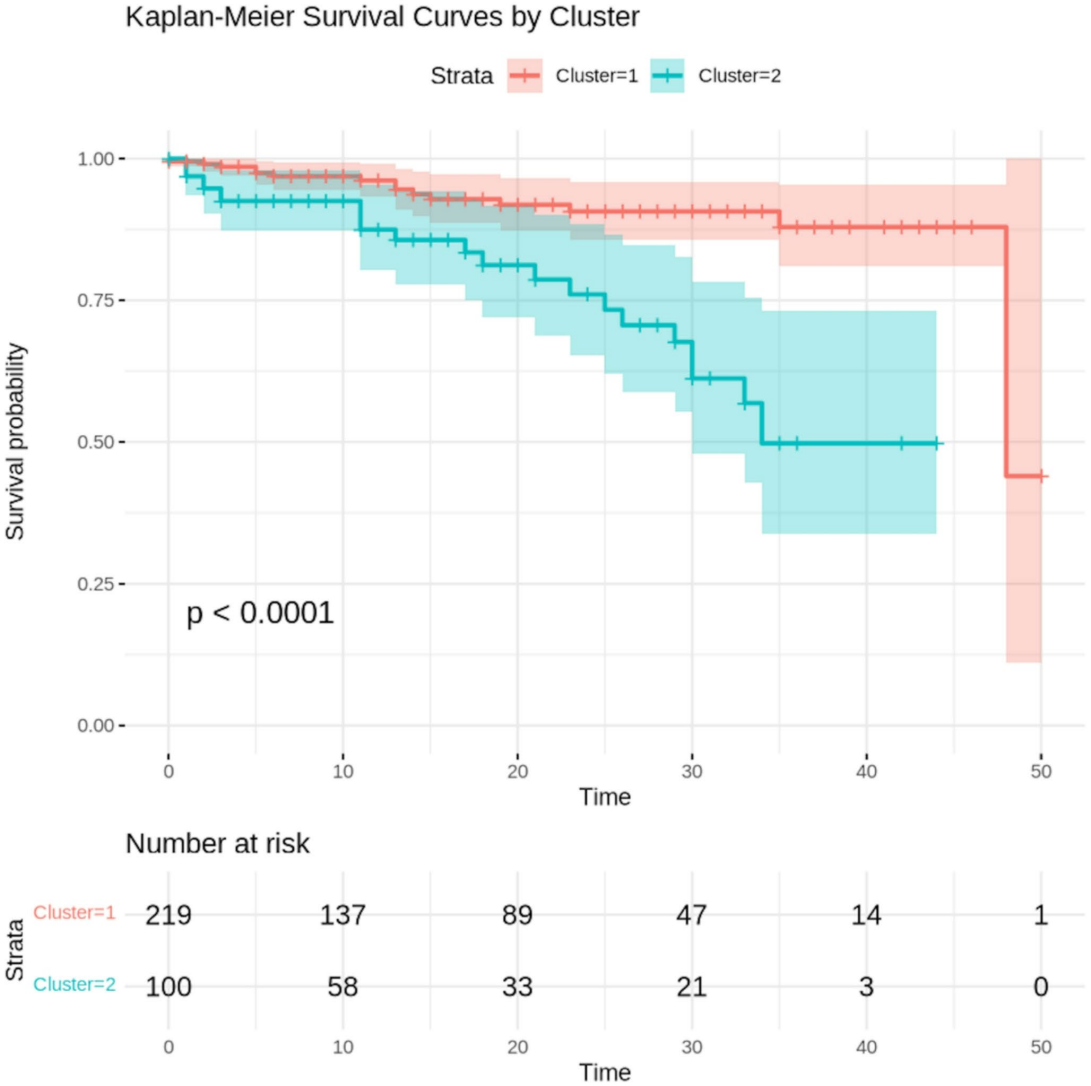
Kaplan-Meier plot for the study endpoint according to clusters. This curve illustrates the time-to-event analysis for the primary composite endpoint (cardiovascular death, aborted SCD, appropriate ICD therapy, HF hospitalisation, LVAD implantation, or heart transplantation). The survival probability is plotted over time for each cluster. The clear separation between the curves (Log-rank *p* < 0.001) indicates a significantly worse prognosis for patients in Cluster 2 compared to those in Cluster 1. The number of patients at risk at specific time points is provided in the table below the graph

**Fig. 4:**
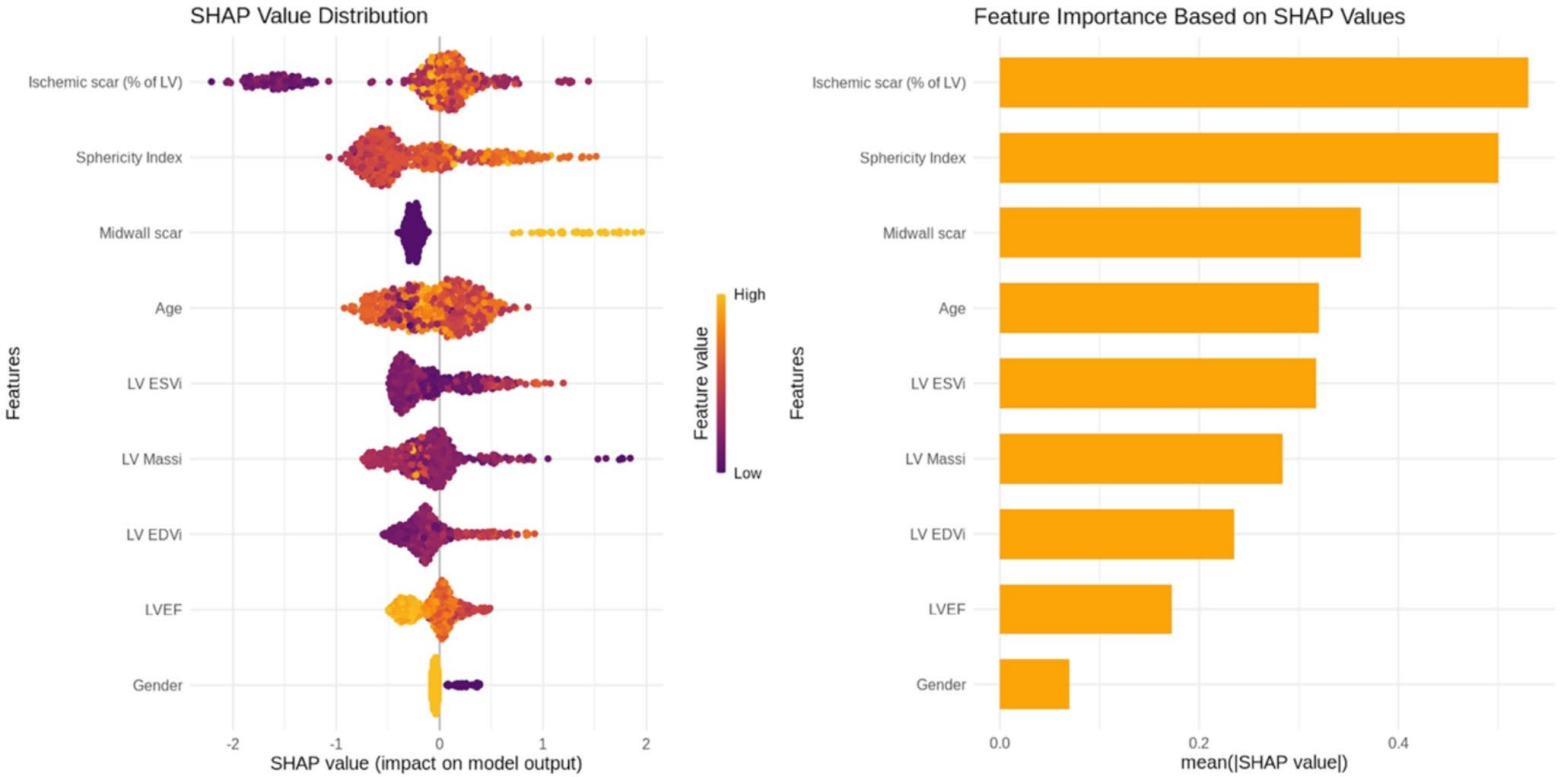
SHapley Additive exPlanations (SHAP) summary plot. The beeswarm plot (left panel) illustrates in detail how each feature’s SHAP values (positive or negative) affect the model’s predictions, highlighting both the size and direction of effects. On the x-axis, each point represents a patient. The position of the point shows whether the feature value for that patient increased the model prediction towards a higher risk (positive SHAP value) or a lower risk (negative SHAP value). The colour indicates the actual feature value for that patient. The bar plot (right panel) presents the mean absolute SHAP values, ranking features vertically from most to least important in predicting the composite outcome

Univariate Cox proportional hazards regression analysis revealed that patients in Cluster 2 had a significantly higher risk of experiencing the composite outcome compared to those in Cluster 1 (HR = 3.96, 95% CI: 2.02–7.76, *p* < 0.001). Kaplan-Meier curves (Fig. 3) show the difference in survival outcomes between the two clusters, with Cluster 2 showing worse survival than Cluster 1 (log-rank *p* < 0.001). During follow-up, 20 patients experienced non-cardiovascular death. CIFs of the composite outcome increased over time in both clusters and were consistently higher in Cluster 2; at 12 months, the CIF was 3.2% in Cluster 1 compared to 10.2% in Cluster 2; at 24 months, 6.5% versus 16.7%; and at 48 months, 21.3% versus 29.0%, respectively. The subdistribution hazard ratio (SHR) for the composite outcome in Cluster 2 versus Cluster 1 was 3.48 (95% CI 1.81–6.71, *p* < 0.001), indicating that non-CV death did not materially affect the prognostic associations. SHAP analysis (Fig. 4) provided insights into the contribution of individual variables to the composite outcome, with ischemic scar (% of LV mass), sphericity index and presence of midwall scar being the most influential features.

## Discussion

The study employs unsupervised machine learning to identify distinct phenotypic subgroups in patients with ICM using CMR-derived variables. The results illustrate the usefulness of clustering techniques in revealing clinically meaningful subgroups that differ notably in cardiac function, structural remodelling, fibrotic changes, and ultimately prognosis. These findings mark a significant step forward in understanding ICM heterogeneity, as the identified phenotypes probably reflect different pathophysiological pathways that go beyond traditional risk stratification methods.

### Identification of phenotypic subgroups

Few recent studies have pioneered the use of unsupervised machine learning to identify distinct subgroups of patients with heart failure and cardiomyopathy, offering valuable insights into disease heterogeneity and prognostic stratification.

Verdonschot et al. [15] investigated phenotypic clustering in dilated cardiomyopathy, identifying four subgroups with notable differences in underlying pathophysiology and outcomes. They emphasised the importance of combining genetic, imaging, and clinical data to better understand disease mechanisms and customise treatment approaches.

Shah et al. [16] and Segar et al. [17] investigated the use of phenomapping in the context of heart failure with preserved ejection fraction, identifying three distinct phenotypic subgroups with varying clinical characteristics, therapeutic responses, and outcomes. These studies highlighted the potential of machine learning to uncover hidden patterns within complex datasets, paving the way for more personalised approaches to managing heart failure with preserved ejection fraction.

In the context of ICM, Kwon et al. [18] recently established the usefulness of CMR-enriched phenomapping to identify clusters with varying survival rates following surgical revascularisation and surgical mitral valve intervention. This work demonstrated that comprehensive CMR-based phenotyping may aid personalised risk prediction and therapeutic decision making.

Contemporary research has further confirmed the importance of detailed scar characterisation in ICM prognosis. The recently developed CMR-LGE score, based on a large multicentre ICM cohort (*n* = 3,591), showed that combining LGE extent, location, transmurality, and midwall fibrosis performs well, with higher prognostic value than traditional prognosticators in predicting mortality [19]. Furthermore, advancements in automated CMR analysis using deep learning have made comprehensive phenotyping more practical for clinical use, with automated volumetric and scar quantification providing comparable prognostic value to manual measurements while allowing large-scale application [20, 21].

The current study enhances and broadens existing knowledge by using unsupervised clustering to identify distinct phenotypic subgroups within ICM, further confirming the usefulness of CMR in understanding disease heterogeneity. The KAMILA clustering algorithm successfully detected two distinct phenotypes among 319 ICM patients. Cluster 1, consisting of 219 patients, was characterised by better cardiac function, including higher left ventricular ejection fraction (LVEF), smaller ventricular volumes, and lower indexed LV mass. This phenotype represents a compensated form of ICM where myocardial remodelling has not yet advanced, potentially reflecting either limited initial ischemic injury or effective adaptive mechanisms. Conversely, Cluster 2, comprising 100 patients, displayed features of a more advanced disease stage, including lower LVEF, larger ventricular volumes, higher indexed LV mass, and a greater prevalence of midwall fibrosis. This phenotype exemplifies advanced ICM with extensive structural and functional decline, suggesting ongoing maladaptive remodelling processes that align with recent findings on the prognostic significance of midwall fibrosis in ICM populations [22, 23]. The distinct separation of clusters in principal component analysis (PCA) and the high proportion of variance explained (62.6%) further validate the robustness of the identified phenotypes and imply that these represent unique biological entities rather than arbitrary groupings.

The differences between the clusters go beyond traditional risk markers, such as LVEF, to include parameters like the sphericity index and midwall fibrosis. The sphericity index, which measures ventricular geometry, was notably higher in Cluster 2, indicating more marked adverse remodelling. This finding is particularly significant given the evidence that geometric remodeling parameters provide incremental prognostic value beyond traditional volumetric measurements [24, 25]. Likewise, the higher occurrence of midwall fibrosis in Cluster 2 highlights the importance of myocardial tissue characterisation in risk stratification [19, 22, 23].

These findings emphasise the potential of unsupervised clustering to integrate multidimensional data and uncover new insights into disease heterogeneity; additionally, they surpass previous phenomapping studies by providing a validated, interpretable framework for personalised risk stratification that could inform therapeutic decisions and the selection of patients for advanced interventions.

### Prognostic implications

The prognostic significance of the identified clusters was clear in the univariate Cox regression analysis and Kaplan-Meier survival curves. Patients in Cluster 2 faced nearly four times the risk of experiencing the composite outcome (cardiovascular death, aborted sudden cardiac death, appropriate implantable cardioverter-defibrillator therapy, heart failure hospitalisation, LV assist device implantation, and heart transplantation) compared to those in Cluster 1. This notable difference in outcomes highlights the clinical importance of the clustering approach and its potential to improve risk stratification in ICM.

The SHAP analysis further clarified the relative importance of individual variables in predicting the composite outcome. Ischemic scar burden (expressed as a percentage of LV mass), sphericity index, and midwall fibrosis emerged as the most influential features, consistent with their established roles in adverse remodelling and arrhythmogenesis [5, 22]. These findings suggest that integrating tissue characterization and geometric parameters into risk models could enhance prognostic accuracy and guide personalised treatment strategies.

### Methodological considerations and study limitations

The use of the KAMILA algorithm is a methodological strength of this study. Unlike traditional clustering methods, KAMILA can handle mixed data types (continuous and categorical variables) without requiring data transformation, thus preserving the original scale of the variables [6]. This is especially beneficial in clinical datasets, where both types of variables are commonly present. The choice of a two-cluster solution was supported by multiple validation methods, including silhouette analysis, within-cluster sum of squares, and gap statistics, ensuring the robustness of the clustering results.

However, the study has several significant limitations that should be recognised. First, the relatively small sample size and brief follow-up may restrict the statistical power to detect subtle phenotypic differences and could influence the stability of the clustering results. Larger cohorts and extended follow-up would be necessary to validate these findings and potentially identify additional phenotypic subgroups. Second, the clustering was conducted on a single-centre cohort, which may limit the generalisability of the findings. External validation in multicentre cohorts is required to confirm the reproducibility of the identified phenotypes. Third, our analysis did not consider the potential impact of different treatment strategies administered during the follow-up period, which could have affected patient outcomes and may confound the prognostic associations observed between clusters. Future studies should incorporate treatment variables as covariates or stratify analyses by therapeutic interventions to better understand the independent prognostic value of phenotypic clustering. Fourth, while the clustering approach offers valuable insights into disease heterogeneity, it does not establish causality. Further research is required to investigate the underlying mechanisms behind the differences observed between clusters. Fifth, we recognise the lack of direct statistical comparison showing that our CMR-based clustering variables outperform conventional clinical variables or established risk prediction models. Conducting comparative analyses against existing prognostic scores would strengthen the evidence for adopting this approach in clinical practice. Finally, the study focused on CMR-derived variables to isolate the value of the morphological, functional, and tissue-based characterisation that CMR provides; this approach allowed defining “pure” imaging phenotypes, which is a crucial first step before integrating them with other data types. The inclusion of additional clinical and laboratory marker variables could further refine the phenotypic subgroups and potentially improve the discriminative performance of the clustering approach [26, 27].

### Clinical implications

The identification of different ICM phenotypes has important clinical implications. Patients in Cluster 2, marked by advanced disease and a poor prognosis, may benefit from more aggressive treatments, such as early consideration of advanced heart failure therapies or increased arrhythmia monitoring, potentially improving outcomes through personalised treatment plans. Conversely, patients in Cluster 1, with better cardiac function and lower risk, may be managed with standard medical therapy, lifestyle changes, and regular monitoring to detect early signs of transition to the high-risk phenotype. Incorporating unsupervised clustering into clinical practice could also support personalised medicine by allowing for tailored treatment approaches based on individual patient phenotypes. For example, the presence of midwall fibrosis or a high sphericity index could lead to closer surveillance for arrhythmias or more aggressive up-titration of heart failure therapies. Moreover, using SHAP values to interpret machine learning models improves transparency and offers clinicians actionable insights into the factors influencing risk predictions [7].

### Future directions

This study establishes a foundation for several future research directions. First, the identified phenotypes should be validated in larger, multicentre cohorts to confirm their generalisability, robustness, reproducibility, and clinical utility before any potential integration into routine practice. Second, including additional data types, such as circulating biomarkers, could further refine the clustering process and offer deeper insights into disease mechanisms. Third, prospective studies are required to assess whether phenotype-guided treatment strategies enhance outcomes in ICM patients. Furthermore, combining chest wall conformation assessment with traditional CMR parameters may yield a more comprehensive phenotypic characterisation and potentially reveal additional clinically relevant subgroups [28]. Lastly, developing user-friendly tools for applying unsupervised clustering within clinical settings could facilitate the translation of these findings into practice. Future research should also aim to understand the temporal evolution of phenotypes and explore the possibility of phenotype transitions over time.

## Conclusions

In conclusion, this study shows how unsupervised machine learning can identify different phenotypic subgroups of ICM patients based on CMR-derived variables. The two clusters found differ notably in cardiac function, structural remodelling, and prognosis, emphasising the potential of clustering methods to improve risk assessment and personalise treatment plans. By combining multidimensional data and using advanced analytical techniques, this research offers a framework to understand the heterogeneity of ICM and to enhance patient outcomes. Future research should focus on confirming these findings, investigating underlying mechanisms, and applying these insights in clinical practice.

## Data Availability

No datasets were generated or analysed during the current study.

## Declarations

### Clinical trial registration

Prospective single-centre observational registry (ClinicalTrials.gov NCT02326324, registration date 22/12/2014).
